# Overexpression of miR‐181a‐5p inhibits retinal neovascularization through endocan and the ERK1/2 signaling pathway

**DOI:** 10.1002/jcp.29733

**Published:** 2020-04-28

**Authors:** Xiuping Chen, Yiyun Yao, Fei Yuan, Bing Xie

**Affiliations:** ^1^ Department of Ophthalmology Zhongshan Hospital of Fudan University Shanghai China; ^2^ Department of Ophthalmology Ruijin Hospital, Shanghai Jiao Tong University School of Medicine Shanghai China

**Keywords:** Endocan, microRNA, oxygen‐induced retinopathy, retinal endothelial cell

## Abstract

Retinal neovascularization (RNV) is a common pathological feature of angiogenesis‐related retinopathy. Endocan inhibition has previously been reported to suppress RNV in oxygen‐induced retinopathy (OIR); however, its molecular mechanisms remain to be elucidated. Here, we investigated the role and mechanism of endocan in OIR. We established an OIR mouse model and detected aberrant *endocan* overexpression in OIR mouse retinas. Endocan inhibition through small interfering RNA or a neutralizing antibody inhibited vascular endothelial growth factor‐induced cell survival, cell proliferation, and tube formation in human retinal endothelial cells in vitro and reduced the RNV area in vivo. Using RNA sequencing, a luciferase reporter assay, and bioinformatics analyses, we identified *endocan* as a microRNA‐181a‐5p target gene. The antiangiogenic effect of miR‐181a‐5p on RNV was verified by intravitreal injection, and we showed that this involved the extracellular signal‐regulated protein kinases 1 and 2 (ERK1/2) signaling pathway. Collectively, our data demonstrate that miR‐181a‐5p/endocan regulates retinal angiogenesis through the ERK1/2 signaling pathway and might represent an attractive therapeutic strategy for RNV.

## Graphical abstract

We identified endocan as a microRNA‐181a‐5p target gene. miR‐181a‐5p/endocan regulates retinal angiogenesis through the extracellular signal‐regulated protein kinases 1 and 2 signaling pathway and might represent an attractive therapeutic strategy for retinal neovascularization.

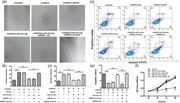

## 1 INTRODUCTION

Retinal neovascularization (RNV) is the most common pathological change in angiogenesis‐related retinopathy, including retinopathy of prematurity (ROP) and proliferative diabetic retinopathy (PDR; Moran et al., 2016), and it can lead to severe visual impairment and even blindness due to leakage and fibrosis of immature blood vessels. Anti‐vascular endothelial growth factor (VEGF) agents are widely used to block RNV and have shown great therapeutic efficacy; however, some patients show a poor or complete lack of response to anti‐VEGF therapy (Dedania & Bakri, 2015; Kruger Falk, Kemp, & Sorensen, 2013). Moreover, VEGF inhibition may result in side effects such as retinal atrophy and tears in the retinal pigment epithelium. It is, therefore, necessary to identify antagonists for other RNV targets that could be used to treat this condition.

Endothelial cell‐specific molecule 1 (endocan or ESM‐1) has been shown to play an important role in the regulation of angiogenesis, endothelial cell activation, and cell adhesion (Rocha et al., 2014; Yilmaz et al., 2014). It is specifically expressed in and secreted by ECs (Bechard et al., 2001) and is highly enriched in retinal endothelial tip cells (del Toro et al., 2010). Previous studies have reported that *endocan* expression is regulated by the proangiogenic factors VEGFA and VEGFC (Rennel et al., 2007; J. W. Shin, Huggenberger, & Detmar, 2008) and is directly correlated with tumor angiogenesis (L. Y. Chen, Liu, Wang, & Qin, 2010; Roudnicky et al., 2013). In our previous study, we found that endocan expression is strongly upregulated in the retina of oxygen‐induced retinopathy (OIR; Su et al., 2018); therefore, we hypothesized that endocan plays an important role in RNV.

MicroRNAs (miRNAs) are small noncoding RNAs that act as critical posttranscriptional regulators, and their major function is to silence target gene expression by translational repression or messenger RNA (mRNA) degradation (Peng & Croce, 2016). Recent studies found that a number of miRNAs are upregulated in the retina of ROP models (Ding et al., 2017; Henn et al., 2019; Yang et al., 2017; Ye, Liu, He, Xu, & Yao, 2014); however, it remains unclear whether other miRNAs that target endocan are involved in RNV development.

In the present study, we screened the miRNA expression profile of OIR mouse retinas using miRNA sequencing analysis and performed bioinformatics analysis to identify potential miRNA response elements in the 3′‐untranslated region (3′‐UTR) of endocan. We also investigated the effects of endocan and these miRNAs on retinal angiogenesis and RNV. We propose that manipulating miR‐181a‐5p/endocan levels may be an attractive therapeutic strategy for treating RNV.

## 2 MATERIALS AND METHODS

### 2.1 Cell culture

Human retinal endothelial cells (HRECs; Angio‐Proteomie, Boston, MA) were cultured in endothelial cell medium supplemented with 5% fetal bovine serum (FBS; Lonza, NJ) and 100 U/ml penicillin–streptomycin in a 5% CO_2_ humidified incubator at 37°C.

### 2.2 Constructs, oligonucleotides, and transfection

Control and endocan‐targeting small interfering RNAs (siRNAs) were synthesized by GenePharma Co. Ltd. (Shanghai, China). AgomiR, agomiR control, miRNA mimic, inhibitor, and nontargeting control oligonucleotides (RiboBio, Guangzhou, China) were transfected into HRECs using riboFECT^TM^ CP Transfection Reagent (RiboBio). Briefly, cells were seeded onto sixwell plates (Corning Inc., Corning, NY) at a density of 5 × 10^4^ cells/ml, transfected with miR‐181a‐5p mimic, miR‐181a‐5p inhibitor, endocan siRNA, or negative control using Lipofectamine 2000 (Invitrogen, Carlsbad, CA) according to the manufacturer's instructions. After 48 hr, cells were treated with rhVEGFA (20 ng/ml) for the indicated time period. Specific sequences are listed in Table S1.

### 2.3 Cell Counting Kit assay

Cell proliferation was assessed using Cell Counting Kit‐8 (CCK‐8; Dojindo Laboratories, Tokyo, Japan), according to the manufacturer's protocol. HRECs were transfected and seeded onto 96‐well plates at a density of 5 × 10^3^ cells/well. After 24, 48, or 72 hr, cells were incubated with fresh medium supplemented with 10% CCK‐8 for 1 hr, and optical density (OD) was measured at 450 nm.

### 2.4 Tube formation assay

Matrigel (BD Biosciences, San Jose, CA) was added to a 48‐well plate (150 μl per well) and allowed to solidify for 30 min at 37°C. Transfected and untransfected HRECs were seeded onto the gel (2 × 10^4^ cells/well) with rhVEGFA (20 ng/ml) as appropriate, and imaged under an inverted microscope after 6 hr. To evaluate capillary‐like structure formation, junctions and meshes were counted as previously described (Feng et al., 2018). Five randomly selected fields from each well were measured using Image‐Pro Plus software (v.6.0; Media Cybernetics Inc., Silver Spring, MA).

### 2.5 Flow cytometry analysis

Apoptosis was detected using an Annexin V‐FITC kit (KeyGen Biotech, Nanjing, China). HRECs were transfected with appropriate constructs or oligonucleotides for 48 hr and cultured with VEGFA (20 ng/ml) for 12 hr. The cells were then harvested, washed, incubated with annexin V‐fluorescein isothiocyanate (FITC) and propidium iodide (PI) for 15 min in the dark, and immediately analyzed using a BD FACSCalibur flow cytometry system (BD Biosciences).

### 2.6 Establishment of an oxygen‐induced ischemic retinopathy mouse model

OIR was induced as previously described (Connor et al., 2009; Su et al., 2018). Briefly, postnatal Day 7 (P7) C57BL/6J mice were exposed to 75% oxygen with their nursing mother for 5 days and returned to normal air (~21% oxygen) at P12 to receive appropriate treatments. Age‐matched control mice were maintained in normal air. Retina samples were collected at P15 or P17.

### 2.7 RNA sequencing analysis

Total RNA was extracted from OIR and normal retina samples using TRIzol reagent (Thermo Fisher Scientific, Waltham, MA) and an miRNeasy Kit (Qiagen, Germantown, MD) and used to prepare an RNA sequencing library. Sequencing was performed on an Illumina HiSeq 2000 sequencing system (Illumina, San Diego, CA), and differentially expressed miRNAs were screened using a fold change threshold value ≥ 1.5, and *p* < .05. A bioinformatics search was performed using TargetScan (http://www.targetscan.org) to predict miRNAs with potential sites of interaction with endocan.

### 2.8 Luciferase reporter assay

Regions of the 3′‐UTR containing predicted endocan binding sites were cloned into the *Xho*I and *Not*I sites of a pmiR‐RB‐REPORT^TM^ vector (RiboBio). Constructs were verified using DNA sequencing. Primers used to amplify wild‐type (WT) and relevant mutant control (MUT) 3′‐UTRs are listed in Table S2. WT or MUT versions of the endocan 3′‐UTR were cotransfected with the pmiR‐RB‐REPORT^TM^ plasmid (RiboBio) into HRECs with an endocan‐regulating miRNA mimic or miR‐NC oligonucleotides using Lipofectamine 2000 (Thermo Fisher Scientific). Cells were cultured for 48 hr and luciferase activity was measured using a Dual‐Glo Luciferase assay system (Promega, Madison, WI). Firefly luciferase activity was normalized to *Renilla* luciferase activity.

### 2.9 Intravitreal injection of neutralizing antibody and miRNA

Mice received an intravitreal injection of miR‐181a‐5p agomir or agomir control miRNA (1 nM; RiboBio) according to the manufacturer's instructions, or with 1 μl mouse endocan neutralizing antibody (NAb; R&D Systems, Minneapolis, MN) at a concentration of 0.5 μg/μl or immunoglobulin G (IgG) isotype Ab, as previously described (Su et al., 2018). After returning to normal air at P12, mice were injected with 1 μl phosphate‐buffered saline (PBS), miR‐NC or miR‐181a‐5p mimic oligonucleotides, endocan Ab, and IgG isotype Ab (*n* = 6 per group). Mice were euthanized at P17. One eye was prepared for immunofluorescent flat‐mount analysis, while the contralateral eye was processed for real‐time quantitative polymerase chain reaction (qPCR) and western blot analysis.

### 2.10 Immunofluorescence

Eyeballs from control and OIR mice were dissected and rapidly frozen in embedding medium (Sakura Finetek, Torrance, CA). Retina sections (10‐μm thick) were thawed, air‐dried, and fixed in 4% paraformaldehyde at room temperature for 10 min. After blocking with 10% FBS in PBS for 1 hr, sections were incubated with goat anti‐mouse endocan Ab (R&D Systems) overnight at 4°C, followed by incubation with an Alexa Fluor 555‐conjugated donkey anti‐goat secondary Ab (1:500; Invitrogen) and FITC‐labeled isolectin B4 (1:50; Vector Laboratories Inc., Burlingame, CA) for 1 hr at room temperature. Sections were then rinsed in PBS and stained with DAPI (Beyotime Biotechnology, Shanghai, China) for 5 min. Images were captured using a fluorescence microscope (Carl Zeiss Microscopy, Thornwood, NY).

### 2.11 Retina flat‐mount analysis

OIR and control mice that received intravitreal injections at P12 were euthanized at P17. Enucleated eyes were fixed with 4% paraformaldehyde for 4 hr then blocked with PBS containing 0.1% Triton X‐100 and 0.5% bovine serum albumin for 1 hr. Retinas were harvested and stained with FITC‐labeled isolectin B4 for 45 min then washed in PBS, cut into 4–6 radial petals, flat‐mounted with fluorescence mounting medium (DAKO; Agilent Technologies, CA), and sealed with a cover slip. Images were acquired using a fluorescence microscope and merged to show the entire retina using Photoshop CS 6.0 software (Adobe Systems, San Jose, CA). Neovascular areas were quantified by a blinded investigator using imaging software (Image Pro Plus; Media Cybernetics Inc., Rockville, MD).

### 2.12 Quantitative real‐time PCR

Total RNA was extracted from HRECs or retinas using TRIzol reagent (Thermo Fisher Scientific) according to the manufacturer's protocol, and 1 μg was reverse‐transcribed into complementary DNA (cDNA) using an M‐MLV Reverse Transcriptase System (Thermo Fisher Scientific). Real‐time qPCR was conducted in 10 μl total volume with SYBR Green Master Mix using a LightCycler 480 Real‐Time System (Roche, Mannheim, Germany). Cyclophilin A was used as an internal control. For miRNA detection, total cDNA was synthesized using an miRNA first strand cDNA synthesis kit (Sangon Biotech, Shanghai, China), and real‐time qPCR was performed using a miScript SYBR Green PCR Kit (Qiagen). U6 small nuclear RNA was used as an internal control. Specific sequences are listed in Table S1. Expression levels were quantified using the $2^{-\Delta \Delta C_{t}}$ method (Lewis & Rice, 2016).

### 2.13 Western blot analyses

Western blot analyses were performed as previously described (X. P. Chen et al., 2016) with the following primary antibodies: anti‐endocan (0.1 μg/ml; R&D Systems); anti‐ERK1/2 (1:1,000; Cell Signaling Technology); anti‐p‐ERK1/2 (1:1,000; Cell Signaling Technology); anti‐VEGF (1:1,000; Abcam); anti‐VEGFR1 (1:1,000; Abcam); anti‐VEGFR2 (1:1,000; Cell Signaling Technology); and GAPDH (1:1,000; Cell Signaling Technology).

### 2.14 Statistical analysis

Multiple comparisons were conducted using one‐way analysis of variance followed by Bonferroni's or Dunnett's post hoc tests. Comparisons between two groups were conducted using Student's *t* tests. All data are expressed as mean ± standard error of the mean of at least three independent experiments. All statistical analyses were performed using SPSS 22.0 software (Chicago, IL). *p* < .05 were considered statistically significant.

## 3 RESULTS

### 3.1 Endocan is highly expressed in OIR mouse model retinas

**Figure 1:**
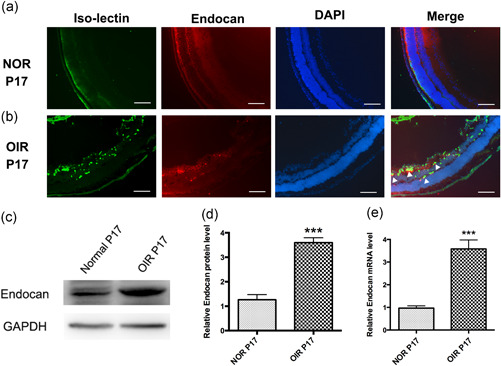
Endocan is highly expressed in OIR mouse model retinas. (a and b) Immunofluorescent stained ocular frozen sections from the OIR mice model with anti‐endocan antibody (red) and isolectin B4 (green). Normal age‐related mice were used as control. White arrowheads in the merged image indicated the colocation between endocan and isolectin B4. Scale bars = 50 μm. (c and d) Western blot of endocan protein in OIR models at P17. GAPDH was used for equal protein loading. (e) Real‐time qPCR analysis of endocan mRNA in the retinas in OIR mice at P17, which were endogenously normalized to GAPDH. Data in graphs presented as mean ± *SEM*. ***p < .001. mRNA, messenger RNA; OIR, oxygen‐induced retinopathy; qPCR, quantitative polymerase chain reaction; *SEM*, standard error of the mean

First, we sought to determine whether endocan expression increased in OIR mouse retinas. Retinas were collected from OIR and normal mice at P17, and immunofluorescent double‐labeling revealed that strong endocan expression colocalized with isolectin B4 (an endothelial cell marker) in the OIR retinas, while endocan staining was faint in the control retinas (Figure 1a,b). Western blot analysis further confirmed that endocan protein levels were significantly higher in the OIR retina at P17 (Figure 1c,d). *Endocan* mRNA levels were measured using real‐time qPCR and were also increased in the OIR retina compared with controls (Figure 1e). These results are consistent with our previous study (Su et al., 2018) and suggest that endocan may play a critical role in retinal angiogenesis.

### 3.2 Effect of endocan siRNA knockdown on VEGF‐induced survival, proliferation, and tube formation of HRECs

**Figure 2:**
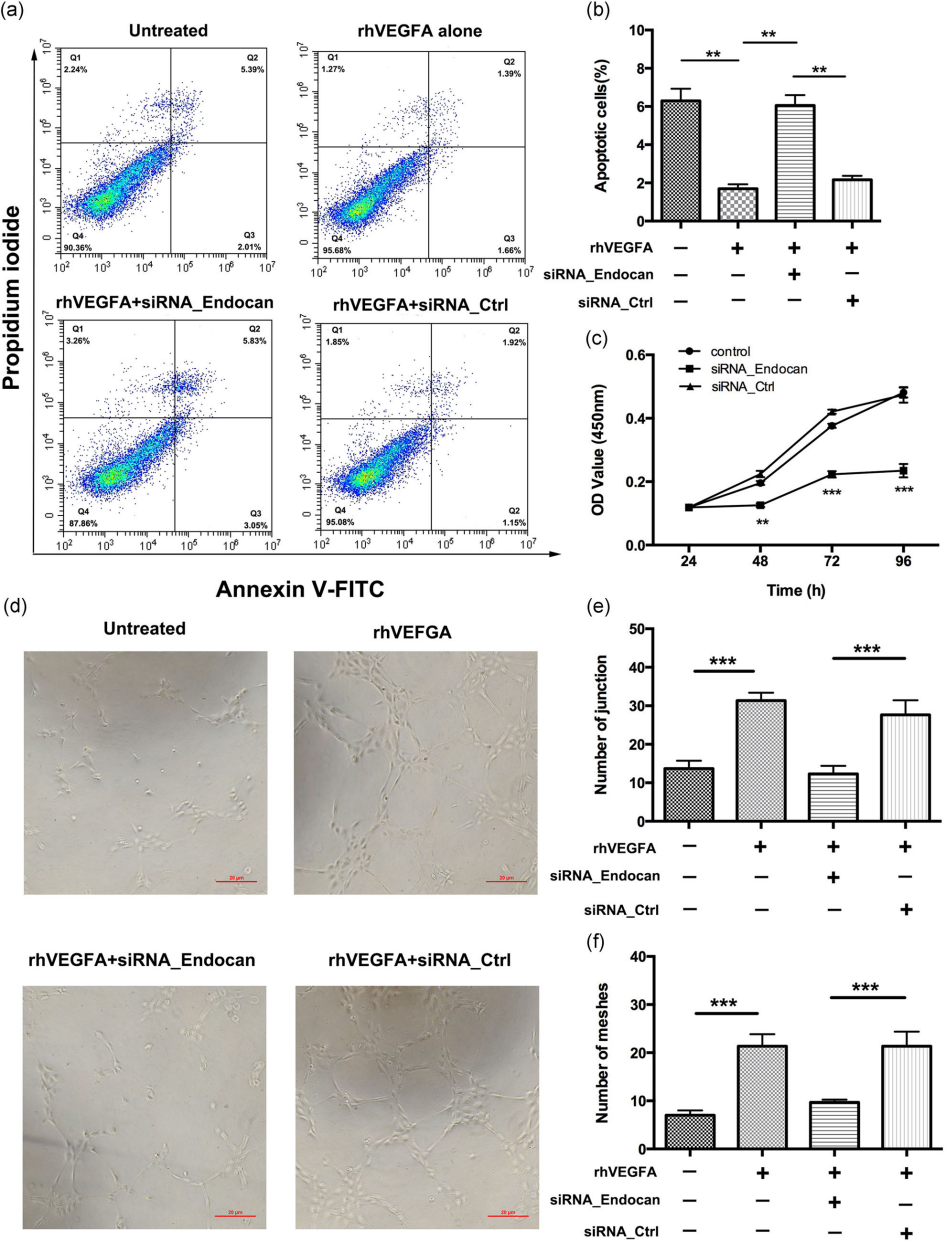
Endocan silence suppresses VEGF‐induced angiogenesis in HRECs. (a and b) HRECs were transfected with siRNA_Endocan or negative control (siRNA_Ctrl) for 48 hr, then exposed to VEGF (20 ng/ml, 24 hr) before apoptosis assay. The percentage of apoptotic cells was determined by annexin V‐FITC/propidium iodide staining and flow cytometry. (c) CCK‐8 was determined with CCK‐8 assay kit and expressed as the percentage of enzyme activity compared with the untreated group (*n* = 5 independent experiments). OD values were significantly reduced in the siRNA‐endocan group compared with those in the control or NC group. (d) HRECs were transfected with siRNA_endocan or negative control (siRNA_Ctrl) for 48 hr, then exposed to rhVEGFA (20 ng/ml, 6 hr), and images were taken under an inverted microscope (*n* = 5 independent experiments). The quantification of junctions (e) and meshes (f) from five randomly selected fields was shown. Scale bars = 20 μm. Data in graphs presented as mean ± *SEM*. **p < .01, ***p < .001. CCK‐8, Cell Counting Kit‐8; FITC, fluorescein isothiocyanate; HREC, human retinal endothelial cell; OD, optical density; *SEM*, standard error of the mean; siRNA, small interfering RNA; VEGF, vascular endothelial growth factor

Next, we investigated the angiogenic effects of endocan using RNA interference technology (Agrawal et al., 2003) to knockdown *endocan* expression. FITC‐conjugated annexin V and PI staining were used to identify apoptotic cells. As shown in Figure 2a,b, VEGF significantly inhibited apoptosis in HRECs compared to untreated controls (1.69 ± 0.15 vs. 6.29 ± 0.31; *p* < .01), and this antiapoptotic effect was significantly attenuated by transfection with siRNA_endocan (6.05 ± 0.37% vs. 2.16 ± 0.16%; *p* < .01).

Next, we conducted CCK‐8 assays to investigate the effect of siRNA_endocan on cell proliferation. OD values in the siRNA‐endocan group were significantly lower than those in the siRNA_control and NC groups from 48 to 96 hr, indicating that inhibiting endocan suppressed cell proliferation in a time‐dependent manner (siRNA_endocan group vs. controls; *p* < .01 at 48 hr, *p* < .001 at 72 and 96 hr; Figure 2c).

To further investigate the angiogenic effects of endocan, we performed a VEGF‐induced tube formation assay using cultured HRECs (Figure 2d). Silencing endocan with siRNA_endocan significantly reduced the number of junctions (12.33 ± 1.59 vs. 27.67 ± 2.01; *p* < .001) and meshes (9.67 ± 0.62 vs. 21.33 ± 2.89; *p* < .001) in tubular structures compared with control siRNA (Figure 2e,f). Taken together, these data suggest that inhibiting endocan can modulate angiogenic activity in vitro.

### 3.3 Neutralizing endocan inhibits RNV in an OIR mouse model

**Figure 3:**
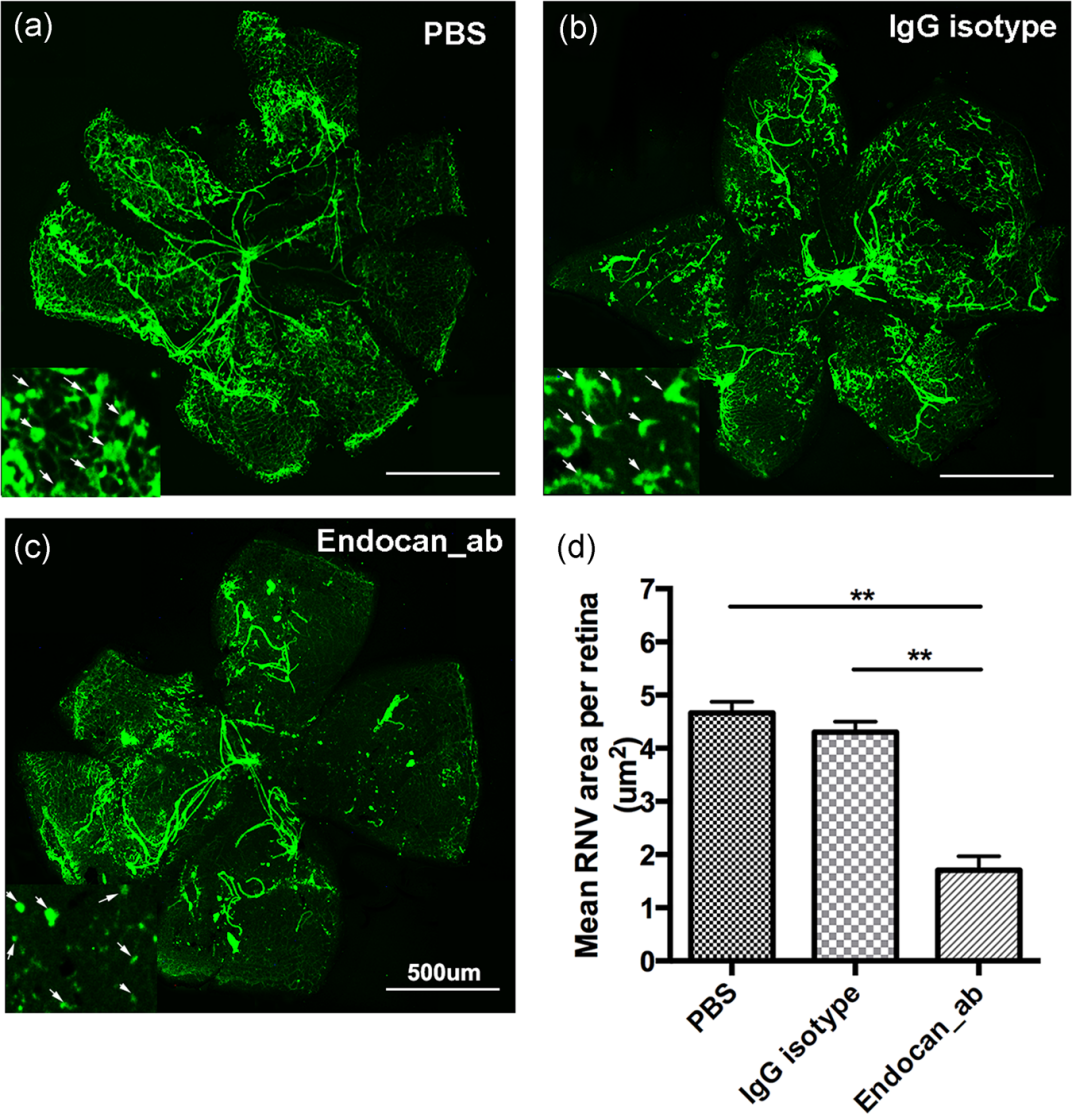
Endocan silence suppresses retinal angiogenesis in vivo. (a–c) Immunofluorescence staining of retinal flat‐mounts of OIR mouse model treated with mouse endocan NAb. Retinas were flat‐mounted and stained with FITC‐lectin at P17 (*n* = 6 mice/group). Scale bars = 500 μm. (d) Quantification of the RNV area (μm^2^) revealed the angiogenic effects of endocan. Statistics were analyzed using one‐way ANOVA with Bonferroni's post hoc test. **p < .01, ***p < .001. ANOVA, analysis of variance; FITC, fluorescein isothiocyanate; NAb, neutralizing antibody; OIR, oxygen‐induced retinopathy; RNV, retinal neovascularization

To investigate the role of endocan in retinal pathological angiogenesis, we used an OIR mouse model, which is a classic model of RNV. Intravitreal injections of endocan Ab (0.5 μg/μl, NAb; R&D Systems), IgG isotype Ab, or PBS control were performed at P12. Endocan_Ab treatment significantly reduced RNV area (Figure 3a–c) and markedly reduced RNV size compared with those in the PBS‐ or IgG isotype‐inoculated groups (4.67 ± 0.21 vs. 4.30 ± 0.20 vs. 1.72 ± 0.26 μm^2^; Figure 3d). These data indicate that blocking endocan can modulate RNV development in vivo.

### 3.4 *Endocan* is a target gene of miR‐181a‐5p

**Figure 4:**
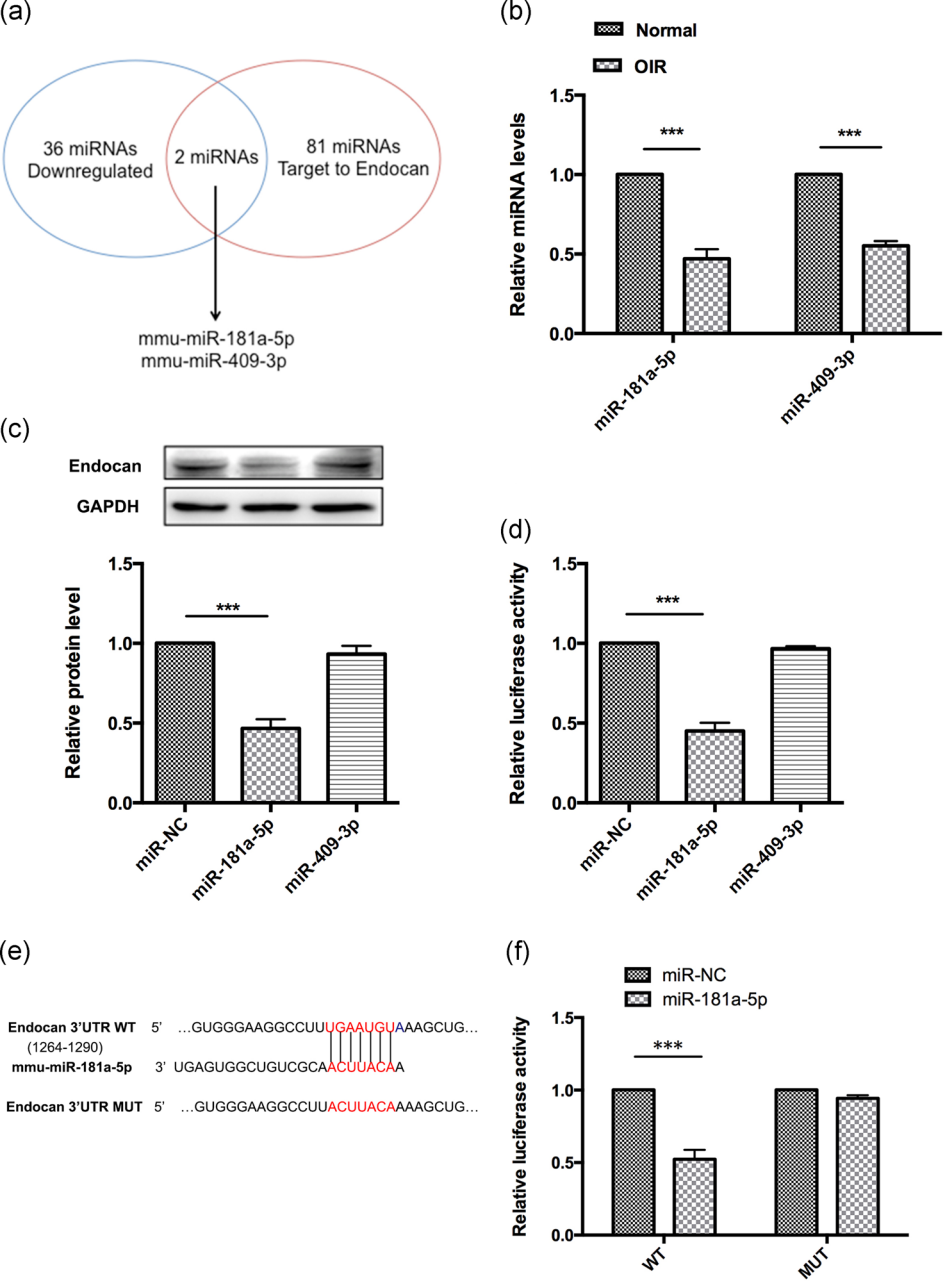
Endocan is a target gene of miR‐181a‐5p. (a) RNA sequencing analysis and bioinformatics predicted two candidate miRNAs targeting endocan 3′‐UTR. (b) Real‐time qPCR detected the expression of miR‐181a‐5p and miR‐409‐3p in normal and OIR groups (*n* = 5/group; Student's *t* tests). Protein expression (c) and luciferase activity (d) of endocan in HRECs after transfection with miR‐181a‐5p mimics, miR‐409‐3p mimics, or miR‐NC were detected. GAPDH was used as an internal control for western blot (*n* = 3 independent experiments; Student's *t* tests). (e) Predicted binding site of miR‐181a‐5p on endocan 3′‐UTR. Red portions of sequences represent the WT and MUT miR‐181a‐5p binding sites in endocan 3′‐UTR. (f) Luciferase activity derived from the indicated 3′‐UTR reporter constructs after cotransfection with miR‐181a‐5p mimic or miR‐NC (*n* = 3 independent experiments; Student's *t* tests). Luciferase activity was normalized by the ratio of firefly and Renilla luciferase signals. **p < .01, ***p < .001 versus the normal or miR‐NC group. 3′‐UTR, 3′‐untranslated region; HREC, human retinal endothelial cell; OIR, oxygen‐induced retinopathy; qPCR, quantitative polymerase chain reaction

To explore the miRNA expression profiles in OIR and normal mouse retinas collected at P15, we performed miRNA sequencing analysis on total RNA from each sample. The cut‐off fold change value was set to 1.5. A total of 36 miRNAs were found to be significantly downregulated (*p* < .05) when RNV tissues were compared to normal tissues (Table S3). Moreover, when we scanned the 3′‐UTR region of endocan using the TargetScan database, we found 81 miRNAs that may target endocan transcripts (Figure 4a). Among these, miR‐181a‐5p and miR‐409‐3p were expressed at a lower level in OIR retinal tissue than in normal tissue. This was confirmed using real‐time qPCR (Figure 4b).

To determine whether *endocan* expression was selectively regulated by these two miRNAs, we transfected HRECs with selected miRNA mimics or miR‐NC. Western blot analysis demonstrated that miR‐181a‐5p suppressed endocan protein expression in HRECs (Figure 4c). This is consistent with results from a dual luciferase reporter assay, which demonstrated that only miR‐181a‐5p directly repressed the endocan 3′‐UTR (Figure 4d). To clarify the link between endocan and miR‐181a‐5p, we constructed luciferase reporter plasmids containing WT endocan or MUT 3′UTR sequences (Figure 4e). miR‐181a‐5p significantly reduced luciferase activity of the WT endocan reporter, but not the endocan‐mut reporter (Figure 4f). Furthermore, the inhibitory effect of miR‐181a‐5p on *endocan* expression was suppressed when putative seed sequences were mutated. Thus, these results demonstrate that miR‐181a‐5p is enriched in the normal mouse retina, is decreased in the pathologic OIR retina, and negatively regulates *endocan* expression by binding to the 3′‐UTR of its mRNA.

### 3.5 miR‐181a‐5p suppresses VEGF‐induced survival, proliferation, and tube formation in HRECs through endocan

**Figure 5:**
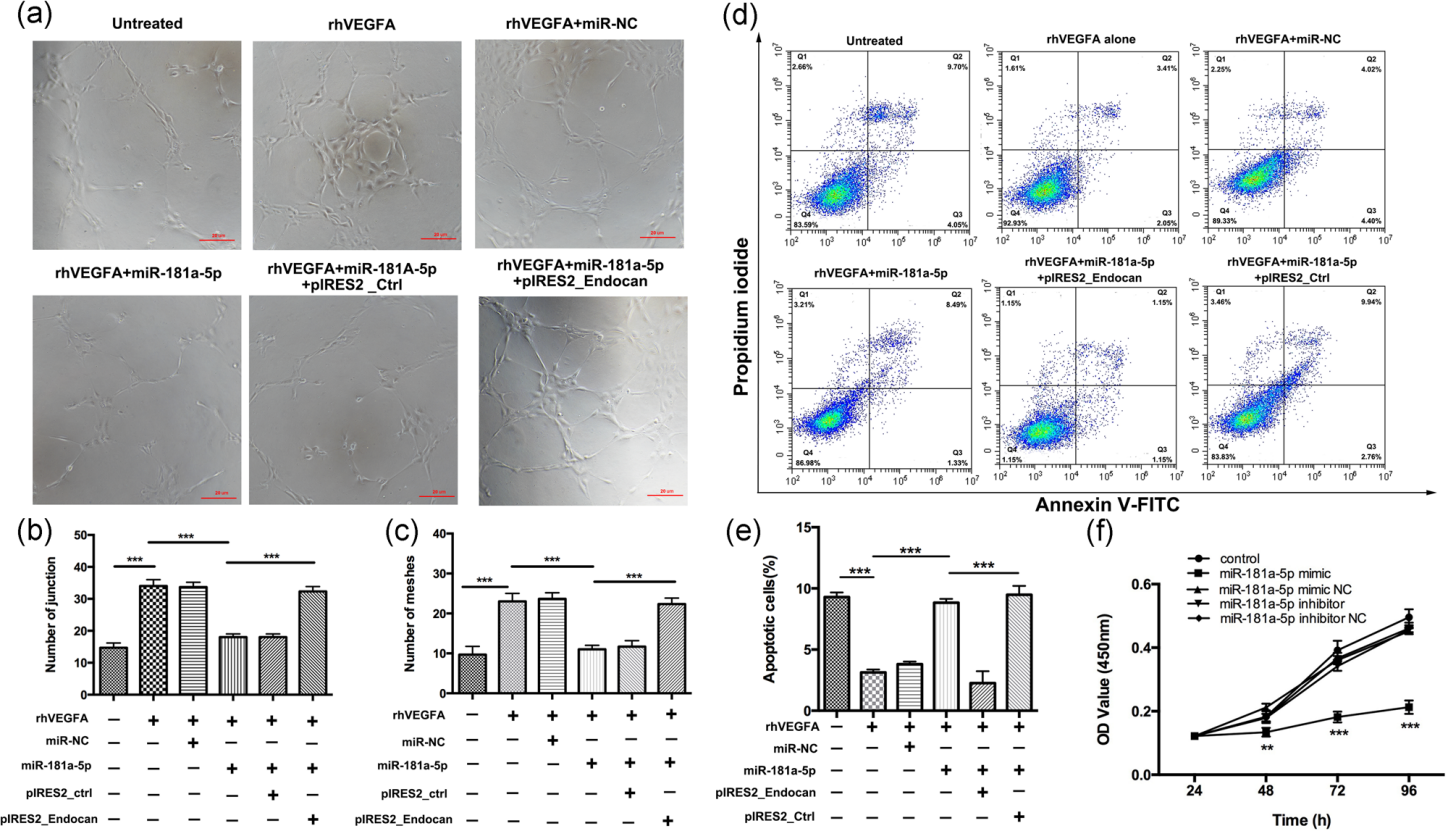
miR‐181a‐5p inhibits VEGF‐induced angiogenesis by targeting endocan in HRECs. (a) HRECs were transfected with miR‐181a‐5p mimic, miR‐NC, pIRES2_endocan, and pIRES2_Ctrl for 48 hr, seeded on Matrigel, and then exposed to rhVEGFA (20 ng/ml, 6 hr) before images were taken using an inverted microscope (*n* = 5 independent experiments). Scale bars = 20 μm. (b and c) The tube formation was quantified by calculating the number of junctions and meshes in each image. (d) HRECs were transfected with miR‐181a‐5p mimic, negative control (miR‐NC), pIRES2_endocan, and pIRES2_Ctrl for 48 hr and then exposed to VEGF (20 ng/ml, 24 hr) before apoptosis assay. (e) The percentage of apoptotic cells was determined by annexin V‐FITC/propidium iodide staining and flow cytometry. (f) CCK‐8 was determined with CCK‐8 assay kit and expressed as the percentage of enzyme activity compared with the inhibitor group and untreated group (C; *n* = 5 independent experiments). OD values were significantly reduced in the miR‐181a‐5p mimic group compared with those in the inhibitor or NC group. Data in graphs represented as mean ± *SEM*. **p < .01, ***p < .001. CCK‐8, Cell Counting Kit‐8; FITC, fluorescein isothiocyanate; HREC, human retinal endothelial cell; OD, optical density; *SEM*, standard error of the mean; VEGF, vascular endothelial growth factor

To clarify the role of miR‐181a‐5p in angiogenesis and confirm that endocan is a functional target of miR‐181a‐5p, we performed a series of in vitro analyses. First, HRECs were transfected with miR‐181a‐5p mimic, miR‐NC, pIRES2_endocan, or pIRES2_Ctrl in the presence of VEGF (20 ng/ml). Transfection with the miR‐181a‐5p mimic significantly reduced the number of junctions (12.33 ± 1.59 vs. 27.67 ± 2.01; *p* < .001) and meshes (9.67 ± 0.62 vs. 21.33 ± 2.89; *p* < .001) compared with miR‐NC (Figure 5a–c). Notably, rescuing *endocan* expression by cotransfection with the miR‐181a‐5p mimic and pIRES2_endocan plasmid reversed the suppressive effects of miR‐181a‐5p on tube formation.

Results from an apoptosis assay revealed that miR‐181a‐5p‐treated cells displayed a higher percentage of apoptotic cells than miR‐NC‐treated cells (8.82% vs. 3.81%; *p* < .001; Figure 5d,e), and this effect was reversed by cotransfection with the miR‐181a‐5p mimic and pIRES2_endocan plasmid. Taken together, these results show that miR‐181a‐5p overexpression negatively regulates angiogenesis by targeting endocan.

We performed a CCK‐8 assay to investigate the effect of miR‐181a‐5p on cell proliferation. OD values in the miR‐181a‐5p mimic group were significantly lower than those in the miR‐181a‐5p inhibitor and NC groups from 48 to 96 hr, indicating that miR‐181a‐5p overexpression suppressed cell proliferation in a time‐dependent manner (miR‐181a‐5p mimic group vs. mimic controls; miR‐181a‐5p inhibitor; inhibitor control; *p* < .001 at 48, 72, and 96 hr; Figure 5f). These data demonstrate that miR‐181a‐5p is necessary and sufficient to suppress HREC networking, apoptosis, and proliferation in vitro.

### 3.6 miR‐181a‐5p restoration suppresses RNV in an OIR mouse model

**Figure 6:**
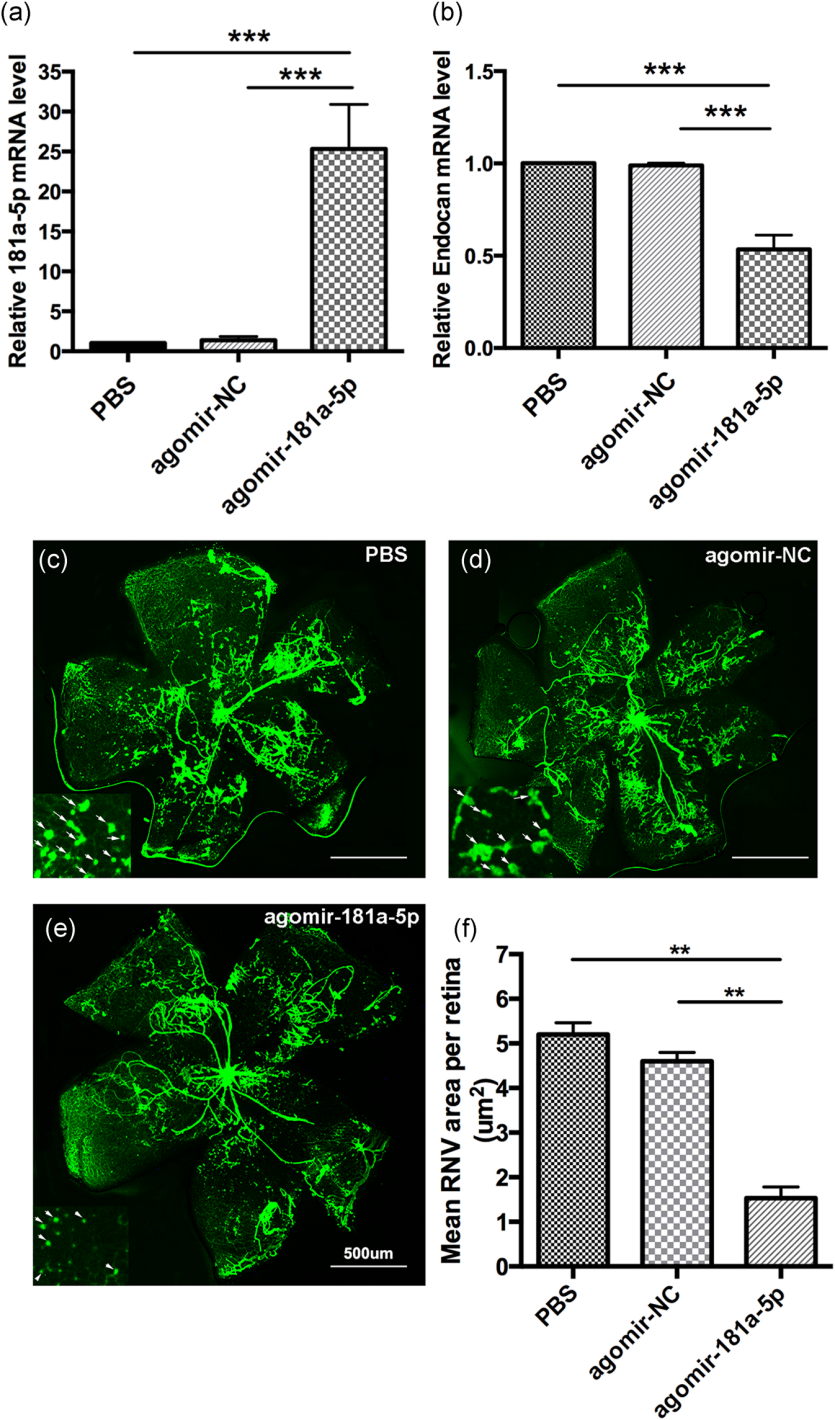
miR‐181a‐5p restoration suppresses RNV in an OIR mouse model. Real‐time qPCR analysis showed upregulation of miR‐181a‐5p (a) and downregulation of endocan (b) by agomiR‐181a‐5p intravitreal injection (*n* = 6 mice/group; representative of three independent experiments). PBS and agomiR‐NC injections were used as controls. Relative mRNA and miRNA levels were normalized to GAPDH or U6 and expressed as the fold change relative to PBS or agomiR‐NC. (c–e) Immunofluorescence staining of retinal flat‐mounts of OIR mouse model treated with agomiR‐181a‐5p. Retinas were flat‐mounted and stained with FITC‐lectin at P17 (*n* = 6 mice/group). (f) Quantification of the RNV area size (μm^2^). Statistics were analyzed using one‐way ANOVA with the least‐significant difference (LSD) method and are representative of three independent experiments. Data in graphs represented as means ± *SEM*. Scale bars = 500 μm. **p < .01, ***p < .001. ANOVA, analysis of variance; FITC, fluorescein isothiocyanate; mRNA, messenger RNA; OIR, oxygen‐induced retinopathy; PBS, phosphate‐buffered saline; qPCR, quantitative polymerase chain reaction; RNV, retinal neovascularization; *SEM*, standard error of the mean

To better understand the therapeutic effects of miR‐181a‐5p on RNV inhibition in vivo, we used an OIR mouse model together with a miR‐181a‐5p agonist, agomiR‐181a‐5p (1 nM) to overexpress miR‐181a‐5p in the eye. AgomiR‐NC was used as a control. Mice were injected at P12 and eyes were collected at P15 for real‐time qPCR, and at P17 for retina flat‐mount assays and western blotting. Real‐time qPCR revealed that miR‐181a‐5p was substantially upregulated and *endocan* mRNA levels were markedly downregulated in agomiR‐181a‐5p‐treated retinas compared with negative controls (Figure 6a,b). These results indicate that agomiR‐181a‐5p was efficiently delivered into the retina by intravitreous injection and that *endocan* is negatively regulated by miR‐181a‐5p overexpression. Quantification of the RNV area revealed that miR‐181a‐5p overexpression reduced RNV by ~65% (4.60 ± 0.20 μm^2^ for agomiR‐NC injection vs. 1.53 ± 0.25 μm^2^ for agomiR‐181a‐5p injection; Figure 6c–f), whereas the agomiR‐NC‐injected group showed no decrease in RNV area compared with the PBS control group. These data are consistent with our in vitro results, and suggest that miR‐181a‐5p inhibits angiogenesis in vivo.

### 3.7 miR‐181a‐5p overexpression suppresses VEGF‐mediated extracellular signal‐regulated kinase pathway activation through endocan

**Figure 7:**
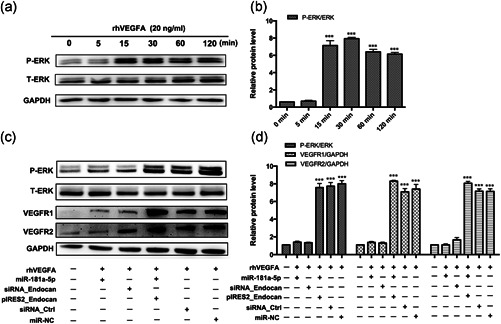
miR‐181a‐5p suppresses VEGF‐mediated activation of ERK1/2 pathways through endocan in HRECs. (a) Western blot analysis of phosphorylated ERK1/2 and total ERK. HRECs were incubated with rhVEGFA (20 ng/ml) for 5, 15, 30, 60, and 120 min in a time‐dependent manner. Phosphorylation of ERK1/2 was detected by western blot analyses. (b) Relative protein levels were normalized to total ERK or GAPDH and expressed as the fold change relative to the untreated group. (c) HRECs were transfected with miR‐181a‐5p mimic or nontargeting mimic control (miR‐NC), siRNA_endocan or negative control (siRNA_Ctrl), or miR‐181a‐5p mimic plus endocan expression plasmid (pIRES2_endocan), then exposed to rhVEGFA (20 ng/ml, 30 min). Phosphorylation of ERK1/2 was detected by western blot. (d) Relative protein levels were normalized to total ERK or GAPDH and expressed as the fold change relative to the untreated group. Data in graphs represent means ± *SEM*. One‐way ANOVA followed by Dunnett's post hoc test was performed for all analyses. **p < .01, ***p < .001 versus the untreated group. ANOVA, analysis of variance; ERK1/2, extracellular signal‐regulated protein kinases 1 and 2; HREC, human retinal endothelial cell; *SEM*, standard error of the mean; siRNA, small interfering RNA; VEGF, vascular endothelial growth factor

The extracellular signal‐regulated kinase (ERK) pathway plays a vital role in modulating angiogenesis (Cai, Xie, Wu, & Wu, 2019; Dai, Gao, Zhao, Wang, & Xie, 2016; Pi et al., 2017). To gain further insight into the mechanism through which miR‐181a‐5p regulates endocan, we sought to identify the mechanism through which this miRNA regulates angiogenesis. On the basis of our previous findings, we focused on the ERK pathway. We first examined extracellular signal‐regulated protein kinases 1 and 2 (ERK1/2) expression levels and phosphorylation status in HRECs treated with VEGFA (20 ng/ml) for different lengths of time (0, 5, 15, 30, 60, or 120 min). Phospho‐ERK1/2 levels increased significantly in a time‐dependent manner, with a peak at 30 min and a decline thereafter (Figure 7a,b). Next, we transfected HRECs with siRNA_endocan or siRNA_Ctrl, miR‐181a‐5p mimic, miR‐NC, or pIRES2_endocan and assayed ERK1/2 phosphorylation. There was a marked reduction in ERK1/2 phosphorylation following *endocan* knockout or miR‐181a‐5p overexpression in HRECs, without a change in total ERK1/2 levels. VEGFR1 and VEGFR2 levels were also decreased. Cotransfection with the miR‐181a‐5p mimic and pIRES2_endocan reversed the changes in P‐ERK1/2, VEGFR1, and VEGFR2 levels. Interestingly, miRNA‐181a‐5p overexpression inhibited VEGFR2 expression more significantly than siRNA_endocan silencing, suggesting that microRNA inhibits target gene expression more efficiently than small interfering RNA (Figure 7c,d). This further confirms the therapeutic potential of miRNA for treating RNV. Collectively, these results indicate that miR‐181a‐5p overexpression suppresses VEGF‐mediated ERK pathway activation by targeting endocan.

## 4 DISCUSSION

RNV involves complex interplay between different cell types, soluble factors, and extracellular matrix components (Byeon et al., 2010). Increasing evidence has shown that multiple miRNAs are important regulators of RNV at the posttranscriptional level; however, their roles in pathological retinal angiogenesis remain poorly understood. In the present study, we found that miR‐181a‐5p was enriched in the normal mouse retina but reduced in the pathological OIR retina. Conversely, *endocan* was highly expressed in the OIR retina, indicating a negative correlation between miR‐181a‐5p and *endocan* expression in OIR. Moreover, we demonstrated that *endocan* was a target gene of miR‐181a‐5p and was downregulated by exogenous miR‐181a‐5p treatment both in vitro and in vivo. Importantly, administration of exogenous miR‐181a‐5p inhibited pathological RNV in OIR and reduced cell survival, proliferation, and tube formation in VEGF‐induced HRECs, suggesting that miR‐181a‐5p has an antiangiogenic role. Finally, overexpression of *endocan* rescued the miR‐181a‐5p‐mediated inhibition of VEGF‐induced HREC survival and tube formation. Together, these findings establish an important role for miR‐181a‐5p/endocan levels in repressing pathological angiogenesis, and suggest that stabilizing miR‐181a‐5p with an intravitreal injection of miRNA with or without antiangiogenic antibodies could be an important therapy for RNV.

*Endocan* expression is positively regulated by VEGF stimulation and shows a direct correlation with tumor angiogenesis (Sagara et al., 2017; Sun et al., 2019). Rocha et al. (2014) demonstrated that *endocan* is strongly expressed in endothelial tip cells in RNV, and may play a critical role in retinal angiogenesis, while Abu El‐Asrar et al. (2015) and Yilmaz et al. (2014) reported that upregulated *endocan* expression in PDR could reflect an association between endothelial cell activation and angiogenesis. Our findings showing increased *endocan* expression in OIR mouse retinas and colocalization with isolectin B4 are consistent with these studies, and suggest a relationship between endocan and RNV development. We also showed that siRNA‐mediated *endocan* silencing abolished VEGF‐induced tube formation and survival in HRECs, and that this was rescued by *endocan* overexpression. Notably, blocking endocan with an intravitreal injection of an endocan NAb suppressed OIR development. Together, these findings indicate that endocan drives angiogenesis both in vivo and in vitro, and it may play a prominent role in RNV‐associated diseases.

miRNAs are importantly involved in eye development, regulation, and angiogenesis (Santulli, 2016; Tiwari, Mukherjee, & Dixit, 2018), with different phenotypes displaying specific miRNA expression profiles (Alberti & Cochella, 2017). We used miRNA sequencing analysis to screen 36 miRNAs that were significantly downregulated in the OIR retina and identified two candidate miRNAs capable of regulating endocan. Western blot and luciferase reporter assays demonstrated that miR‐181a‐5p directly targets the 3′‐UTR of endocan and inhibits its expression. Karali, Peluso, Marigo, and Banfi (2007) demonstrated that microRNA‐181a expression is localized to the ganglion cell layer and innermost layer of the inner nuclear layer, which is consistent with our findings from the current study. Thus, we believe that endocan is a target of miRNA‐181a‐5p. K. H. Shin et al. (2011) showed that miR‐181a exerts tumor‐suppressive effects in oral squamous carcinoma cells, while Li et al. (2015) reported that miR‐181a‐5p inhibits cancer cell migration and angiogenesis. Our finding that miR‐181a‐5p suppresses pathological angiogenesis is consistent with these previous reports and provides evidence that miR‐181a‐5p overexpression dramatically inhibits retinal angiogenesis. Moreover, quantification of the RNV area revealed that miR‐181a‐5p overexpression decreased RNV by ~65% compared with controls, providing additional evidence that miR‐181a‐5p inhibits RNV development. Furthermore, *endocan* rescued the effect of miR‐181a‐5p on tube formation and apoptosis. We also showed that miR‐181a‐5p levels in the retina were substantially upregulated and *endocan* mRNA levels were markedly downregulated compared with controls. These results are consistent with our in vitro findings and indicate that *endocan* is negatively regulated by miR‐181a‐5p. Our data highlight the relationship between miR‐181a‐5p and *endocan*, and suggest that under normal conditions, miR‐181a‐5p is enriched in the retina and maintains vascular homeostasis by repressing *endocan*, its downstream angiogenic target. However, under pathological conditions, downregulated miR‐181a‐5p expression may lead to the upregulation of *endocan*, thus contributing to the development of pathological RNV.

miR‐181a is known to exert a strong antiangiogenic effect on RNV (Yang et al., 2018); however, miR‐181a‐5p target genes and its role in RNV have not yet been fully elucidated. Accumulating evidence suggests that the ERK pathway plays a crucial role in various cellular events, including angiogenesis (Koch, Tugues, Li, Gualandi, & Claesson‐Welsh, 2011; Lemmens, Kusters, Bronckaers, Geurts, & Hendrix, 2017), while endocan deficiency has been shown to decrease p‐ERK1/2 levels (Rocha et al., 2014). Our previous work (Su et al., 2018) suggested that endocan may activate the ERK pathway, therefore, we speculated that the suppression of miR‐181a‐5p‐mediated angiogenesis is dependent on ERK1/2 signaling through the regulation of *endocan* expression. We found that exogenous miR‐181a‐5p treatment or knockout of endogenous *endocan* reduced the expression of VEGFA‐induced phosphorylated ERK1/2 without altering total ERK1/2 protein levels, and decreased VEGFR1 and VEGFR2 levels. Moreover, these effects were reversed by cotransfection with a miR‐181a‐5p mimic and pIRES2_endocan. This suggests that miR‐181a‐5p may be an important regulator of ERK signaling, and that blocking endocan could modulate angiogenic activities during pathological RNV progression by regulating downstream effectors such as p‐ERK1/2, and VEGF family members. Modulating *endocan* expression through miR‐181a‐5p also altered ERK1/2 phosphorylation. Interestingly, miRNA‐181a‐5p overexpression was more effective at silencing *endocan* and inhibiting VEGFR2 expression, suggesting that miRNA can silence target mRNA more efficiently than small interfering RNA. This further confirms the therapeutic potential of miRNA for treating RNV and provides a possible molecular basis for miR‐181a‐5p as an alternative antiangiogenic therapy.

In summary, to the best of our knowledge this study represents the first experimental evidence that miR‐181a‐5p inhibits retinal angiogenesis in vitro and in vivo by directly repressing *endocan*, and provides new evidence that miR‐181a‐5p functions as an angiogenic suppressor. This is particularly important for the treatment of ROP, for which there is currently no approved pharmacological therapy (Beharry, Valencia, Lazzaro, & Aranda, 2016). We also demonstrated that miR‐181a‐5p suppresses RNV by regulating the ERK pathway, providing an important mechanism through which miRNAs regulate angiogenesis. Notably, RNV was not fully inhibited by miR‐181a‐5p transfection, indicating that a combination of anti‐VEGF agents and simultaneous *endocan* blockade may be the optimal therapeutic strategy for RNV. However, further investigation is required to reveal whether miRNA‐181a‐5p and *endocan* activate other signaling pathways during the occurrence and development of RNV, and to determine whether miR‐181a‐5p has other target genes in addition to endocan.

## CONFLICT OF INTERESTS

The authors declare that there are no conflict of interests.

## AUTHOR CONTRIBUTIONS

F. Y. and B. X. conceived and designed the experiments. X. C. and Y. Y. performed the experiments; X. C. drafted the manuscript. X. C. and Y. Y. prepared and reviewed the manuscript. F. Y. and B. X. have given final approval of the version to be published. All authors have read and approved the manuscript.

## ETHICS STATEMENT

All applicable international, national, and/or institutional guidelines for the care and use of animals were followed.

## Supporting information

Supporting information

Supporting information

Supporting information

Supporting information

Supporting information

Supporting information

Supporting information

## Data Availability

Some or all data, models, or code generated or used during the study are available from the corresponding author upon request.
