# Supplementary figures and images for: Overexpression of miR‐181a‐5p inhibits retinal neovascularization through endocan and the ERK1/2 signaling pathway
Source: J Cell Physiol. 2020 Apr 28;235(12):9323–35. doi: 10.1002/jcp.29733 (PMC7587009; doi:10.1002/jcp.29733)

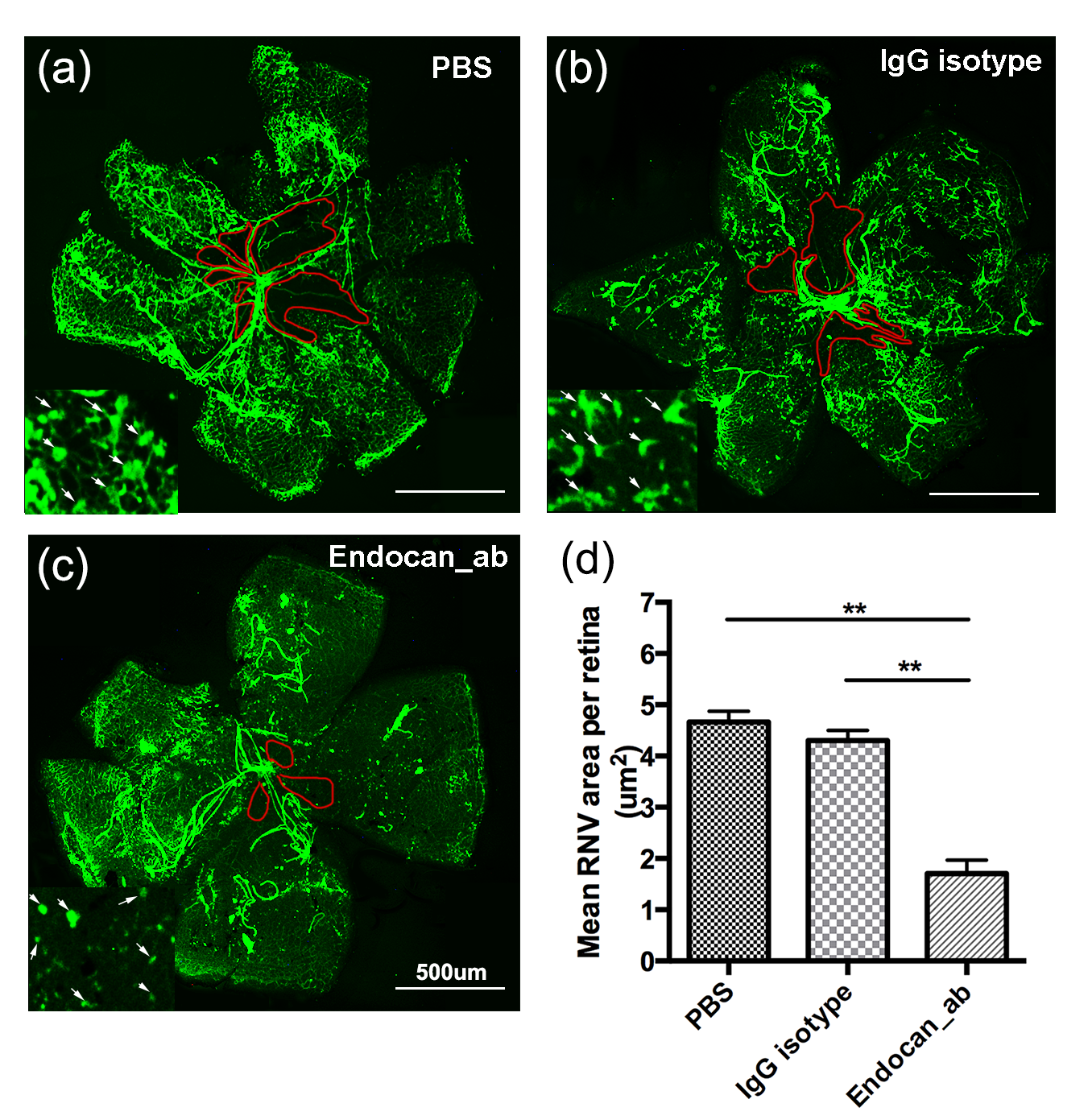

Supplement: Supplementary file 1 — Supporting information [file JCP-235-9323-s001.tif]

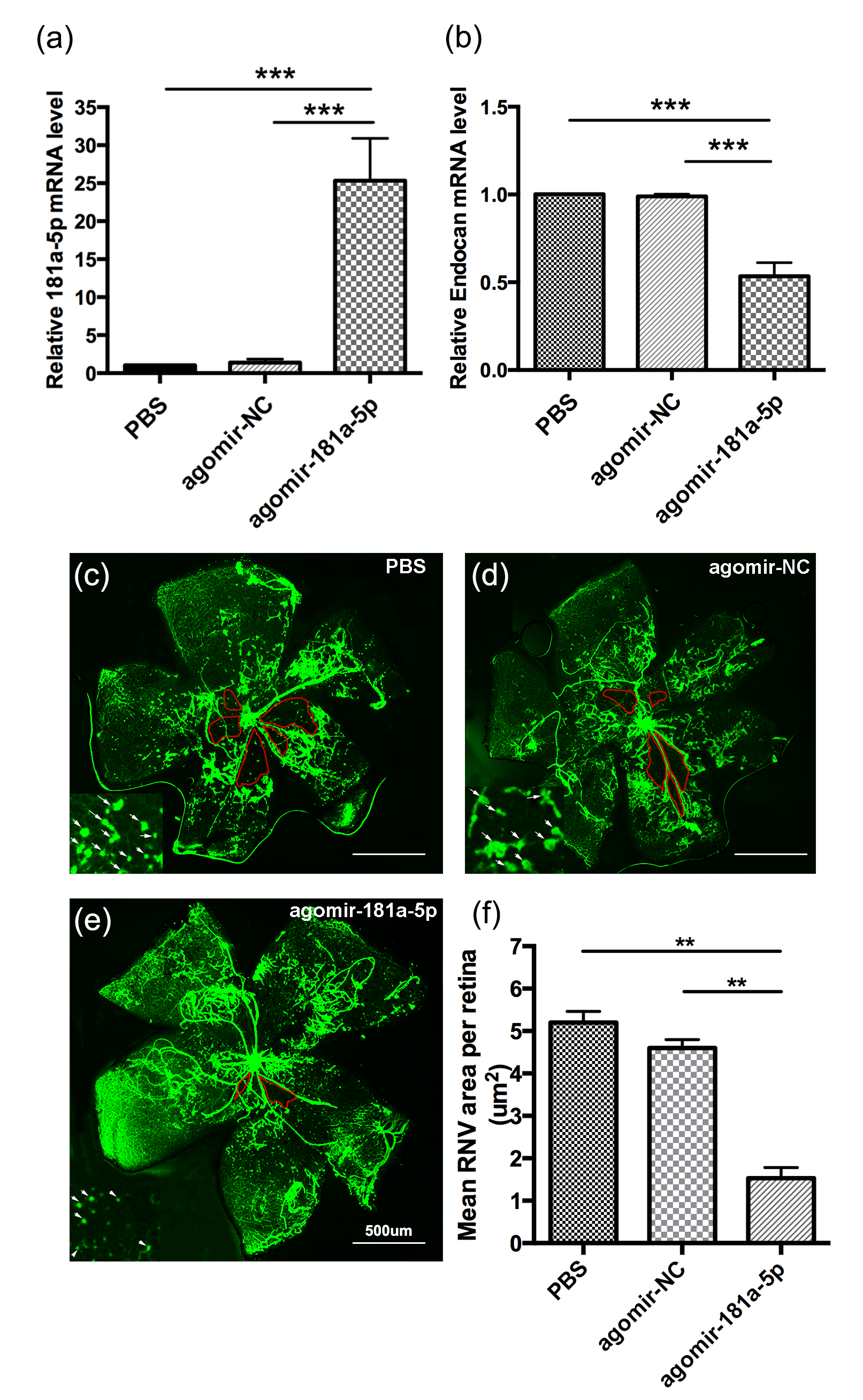

Supplement: Supplementary file 2 — Supporting information [file JCP-235-9323-s002.tif]

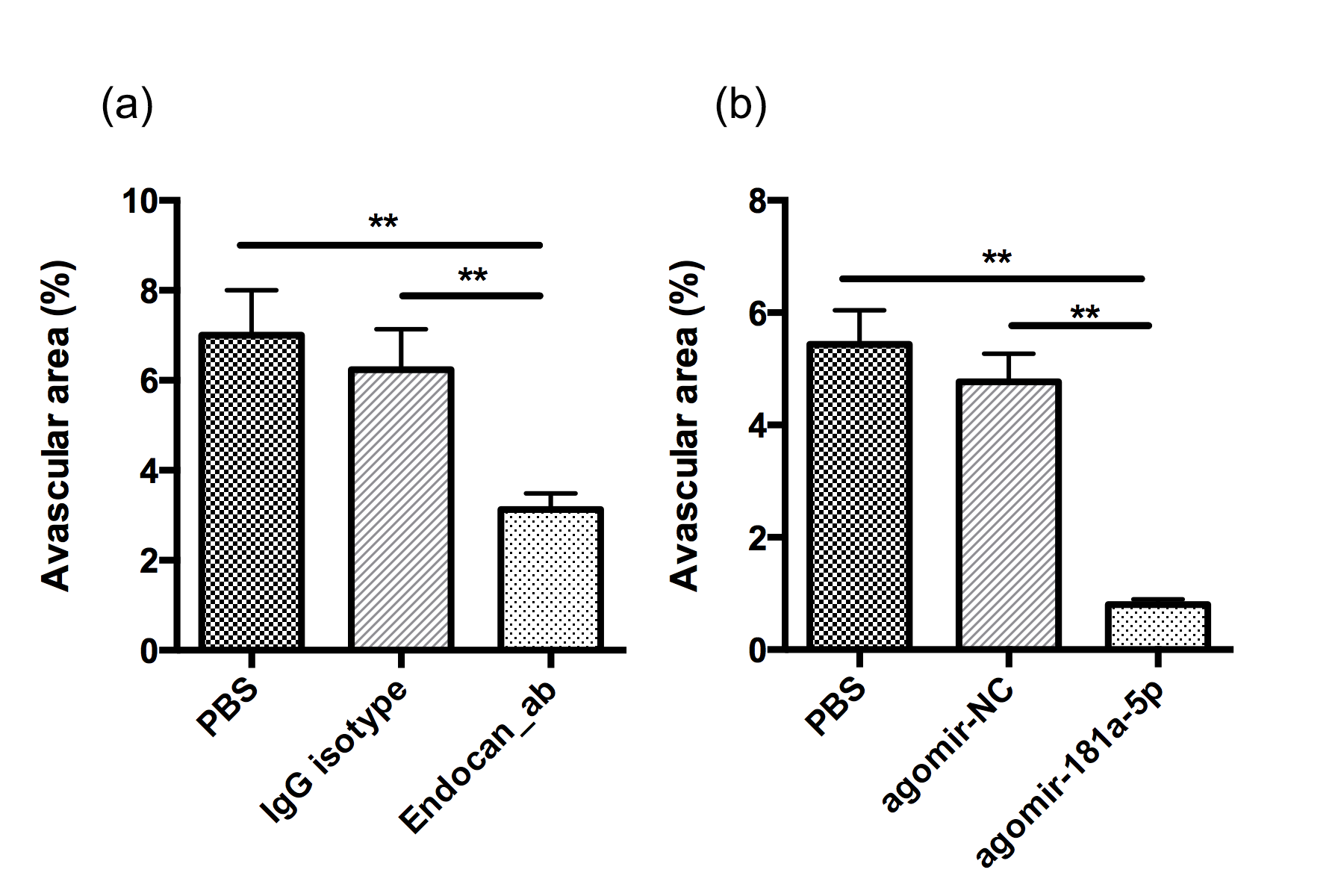

Supplement: Supplementary file 3 — Supporting information [file JCP-235-9323-s003.tif]

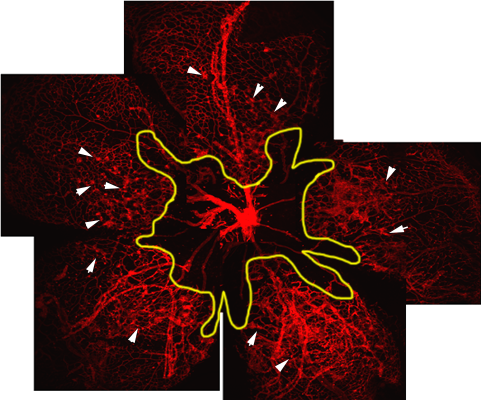

Supplement: Supplementary file 4 — Supporting information [file JCP-235-9323-s004.tif]
