# Supplementary material for: Overexpression of miR‐181a‐5p inhibits retinal neovascularization through endocan and the ERK1/2 signaling pathway
Source: J Cell Physiol. 2020 Apr 28;235(12):9323–35. doi: 10.1002/jcp.29733 (PMC7587009; doi:10.1002/jcp.29733)
Supplement: Supplementary file 5 — Supporting information [file JCP-235-9323-s005.docx]

|  | sense 5‘-3’ | antisense 5‘-3’ |
| --- | --- | --- |
| miRNA mimic |  |  |
| mmu-miR-181a-5p | AACAUUCAACGCUGUCGGUGAGU | ACUCACCGACAGCGUUGAAUGUU |
| mmu-miR-409-3p | GAAUGUUGCUCGGUGAACCCCU | AGGGGUUCACCGAGCAACAUUC |
| mimic NC | UUUGUACUACACAAAAGUACUG | CAGUACUUUUGUGUAGUACAAA |
| miRNA inhibitor |  |  |
| mmu-miR-181a-5p | ACUCACCGACAGCGUUGAAUGUU | CAGUACUUUUGUGUAGUACAAA |
| inhibitor NC | CAGUACUUUUGUGUAGUACAAA |  |
|  |  |  |
| siRNA_Endocan | GGUGAAGAGUUUGGUAUCUTT | AGAUACCAAACUCUUCACCTT |
| siRNA-NC | UUCUCCGAACGUGUCACGUTT | ACGUGACACGUUCGGAGAATT |

Supplementary Table 1. Sequence of miRNA and siRNA
