# Supplementary material for: Overexpression of miR‐181a‐5p inhibits retinal neovascularization through endocan and the ERK1/2 signaling pathway
Source: J Cell Physiol. 2020 Apr 28;235(12):9323–35. doi: 10.1002/jcp.29733 (PMC7587009; doi:10.1002/jcp.29733)
Supplement: Supplementary file 6 — Supporting information [file JCP-235-9323-s006.docx]

Supplemental table 2 Primer and agomir

| pcr primer | F | R |
| --- | --- | --- |
| miR-181a-5p | CGAACATTCAACGCTGTCG | AGTGCAGGGTCCGAGGTATT |
| Endocan | GGAGGATGATTTTGGTGACG | CTGTCACATATGCCCGACTG |
| U6 | CGCGAGAGAAGATTAGCATGG | AGTGCAGGGTCCGAGGTATT |
| Luciferase primer | F | R |
| Endocan-3UTR | GCGCTCGAGACTCGTGGGAAGATCCG | AATGCGGCCGCTGGAACAGCTGTGCCATTTTTATTGA |
| Endocan-3UTR-MUT | AAGATCCGACTTACAAAGCGCAACCGGCTGTTA | TTGCGCTTTGTAAGTCGGATCTTCCCACGAGTA |
| agomir | sense 5‘-3’ | antisense 5‘-3’ |
| micrONTM mmu-miR-181a-5p agomir | AACAUUCAACGCUGUCGGUGAGU | ACUCACCGACAGCGUUGAAUGUU |
| micrONTM agomir Negative Control #22 | UUUGUACUACACAAAAGUACUG | CAGUACUUUUGUGUAGUACAAA |
