# Supplementary material for: Overexpression of miR‐181a‐5p inhibits retinal neovascularization through endocan and the ERK1/2 signaling pathway
Source: J Cell Physiol. 2020 Apr 28;235(12):9323–35. doi: 10.1002/jcp.29733 (PMC7587009; doi:10.1002/jcp.29733)
Supplement: Supplementary file 7 — Supporting information [file JCP-235-9323-s007.docx]

Supplemental table 3 Differentially_Expressed_miRNAs

Down regulated miRNAs

| **MATURE-ID** | **MATURE-SEED** | **MATURE-LENGTH** | **MATURE-SEQ** | **MATURE-ACC** |
| --- | --- | --- | --- | --- |
| mmu-miR-26b-5p | UCAAGU | 21 | UUCAAGUAAUUCAGGAUAGGU | MIMAT0000534 |
| mmu-miR-7a-5p | GGAAGA | 23 | UGGAAGACUAGUGAUUUUGUUGU | MIMAT0000677 |
| mmu-miR-181a-5p | ACAUUC | 23 | AACAUUCAACGCUGUCGGUGAGU | MIMAT0000210 |
| mmu-miR-212-5p | CCUUGG | 23 | ACCUUGGCUCUAGACUGCUUACU | MIMAT0017053 |
| mmu-miR-204-5p | UCCCUU | 22 | UUCCCUUUGUCAUCCUAUGCCU | MIMAT0000237 |
| mmu-miR-181d-5p | ACAUUC | 23 | AACAUUCAUUGUUGUCGGUGGGU | MIMAT0004324 |
| mmu-miR-194-5p | GUAACA | 22 | UGUAACAGCAACUCCAUGUGGA | MIMAT0000224 |
| mmu-miR-190b-5p | GAUAUG | 21 | UGAUAUGUUUGAUAUUGGGUU | MIMAT0004852 |
| mmu-miR-107-3p | GCAGCA | 23 | AGCAGCAUUGUACAGGGCUAUCA | MIMAT0000647 |
| mmu-miR-194-5p | GUAACA | 22 | UGUAACAGCAACUCCAUGUGGA | MIMAT0000224 |
| mmu-miR-433-3p | UCAUGA | 22 | AUCAUGAUGGGCUCCUCGGUGU | MIMAT0001420 |
| mmu-miR-127-5p | UGAAGC | 22 | CUGAAGCUCAGAGGGCUCUGAU | MIMAT0004530 |
| mmu-miR-423-5p | GAGGGG | 23 | UGAGGGGCAGAGAGCGAGACUUU | MIMAT0004825 |
| mmu-miR-409-3p | AAUGUU | 22 | GAAUGUUGCUCGGUGAACCCCU | MIMAT0001090 |
| mmu-miR-181b-5p | ACAUUC | 23 | AACAUUCAUUGCUGUCGGUGGGU | MIMAT0000673 |
| mmu-miR-130a-3p | AGUGCA | 22 | CAGUGCAAUGUUAAAAGGGCAU | MIMAT0000141 |
| mmu-miR-484 | CAGGCU | 22 | UCAGGCUCAGUCCCCUCCCGAU | MIMAT0003127 |
| mmu-miR-495-3p | AACAAA | 22 | AAACAAACAUGGUGCACUUCUU | MIMAT0003456 |
| mmu-miR-320-3p | AAAGCU | 22 | AAAAGCUGGGUUGAGAGGGCGA | MIMAT0000666 |
| mmu-miR-423-3p | GCUCGG | 23 | AGCUCGGUCUGAGGCCCCUCAGU | MIMAT0003454 |
| mmu-miR-487b-3p | AUCGUA | 22 | AAUCGUACAGGGUCAUCCACUU | MIMAT0003184 |
| mmu-miR-361-5p | UAUCAG | 22 | UUAUCAGAAUCUCCAGGGGUAC | MIMAT0000704 |
| mmu-miR-22-3p | AGCUGC | 22 | AAGCUGCCAGUUGAAGAACUGU | MIMAT0000531 |
| mmu-let-7e-5p | GAGGUA | 22 | UGAGGUAGGAGGUUGUAUAGUU | MIMAT0000524 |
| mmu-miR-106b-5p | AAAGUG | 21 | UAAAGUGCUGACAGUGCAGAU | MIMAT0000386 |
| mmu-miR-211-5p | UCCCUU | 22 | UUCCCUUUGUCAUCCUUUGCCU | MIMAT0000668 |
| mmu-miR-375-3p | UUGUUC | 22 | UUUGUUCGUUCGGCUCGCGUGA | MIMAT0000739 |
| mmu-miR-369-3p | AUAAUA | 21 | AAUAAUACAUGGUUGAUCUUU | MIMAT0003186 |
| mmu-let-7b-5p | GAGGUA | 22 | UGAGGUAGUAGGUUGUGUGGUU | MIMAT0000522 |
| mmu-miR-129-5p | UUUUUG | 21 | CUUUUUGCGGUCUGGGCUUGC | MIMAT0000209 |
| mmu-miR-129-5p | UUUUUG | 21 | CUUUUUGCGGUCUGGGCUUGC | MIMAT0000209 |
| mmu-miR-novel-chr2_4838 | CUCGCU | 17 | UCUCGCUGGGGCCUCCA | mmu-miR-novel-chr2_4838 |
| mmu-miR-877-5p | UAGAGG | 20 | GUAGAGGAGAUGGCGCAGGG |  |
| mmu-miR-499-5p | UAAGAC | 21 | UUAAGACUUGCAGUGAUGUUU |  |
| mmu-miR-novel-chr3_12435 | CUCGCU | 17 | UCUCGCUGGGGCCUCCA | mmu-miR-novel-chr3_12435 |
| mmu-miR-129-2-3p | AGCCCU | 22 | AAGCCCUUACCCCAAAAAGCAU | MIMAT0000544 |

Up-regulated miRNAs

| **MATURE-ID** | **pre-miRNA arm (5p or 3p)** | **PRE-ID** | **PRE-ACC** | **MATURE-SEED** |
| --- | --- | --- | --- | --- |
| mmu-miR-503-5p | 5p | mmu-mir-503 | MI0003538 | AGCAGC |
| mmu-miR-21a-5p | 5p | mmu-mir-21a | MI0000569 | AGCUUA |
